# High-density lipoprotein cholesterol subfractions HDL2 and HDL3 are reduced in women with rheumatoid arthritis and may augment the cardiovascular risk of women with RA: a cross-sectional study

**DOI:** 10.1186/ar3842

**Published:** 2012-05-14

**Authors:** Elke Arts, Jaap Fransen, Heidi Lemmers, Anton Stalenhoef, Leo Joosten, Piet van Riel, Calin D Popa

**Affiliations:** 1 Department of Rheumatology, Radboud University Nijmegen Medical Centre, Geert Grooteplein 8, 6500 HB Nijmegen, The Netherlands; 2 Department of Internal Medicine, Radboud University Nijmegen Medical Centre, Geert Grooteplein 8, 6500 HB Nijmegen, The Netherlands

## Abstract

**Introduction:**

Higher levels of high density lipoprotein (HDL) subfractions HDL3-chol and particularly HDL2-chol protect against cardiovascular disease (CVD), but inflammation reduces the HDL level and may impair its anti-atherogenic effect. Changed HDL composition through the impact of inflammation on HDL subfractions may contribute to the excess risk of CVD in rheumatoid arthritis (RA). In this study, we investigated whether HDL2-chol and HDL3-chol concentrations differ between RA patients and healthy controls, and whether these levels are related to the level of RA disease activity.

**Methods:**

Non-fasting blood samples were collected from 45 RA patients and 45 healthy controls. None of the participants had a history of CVD, diabetes, or used lipid-lowering drugs. HDL2-chol and HDL3-chol concentrations were obtained by ultracentrifugation. Regression modeling was used to compare HDL subfraction levels between RA patients and healthy controls, and to analyze the effect of disease activity on HDL2-chol and HDL3-chol.

**Results:**

HDL2-chol and HDL3-chol were significantly lower in RA patients compared to healthy controls (*P *= 0.01, *P *= 0.005, respectively). The HDL2:HDL3 ratio was significantly lower in patients compared to controls (*P *= 0.04). Reduced HDL2-chol and HDL3-chol levels were primarily present in female RA patients and not in male RA patients. A modest effect of the disease activity score in 28 joins ( DAS28) on HDL2-chol concentrations was found, after correction for disease duration, glucocorticosteroid use and body mass index (BMI), with a 0.06 mmol/L decrease with every point increase in DAS28 (*P *= 0.05). DAS28 did not significantly affect HDL3-chol concentrations (*P *= 0.186).

**Conclusions:**

Both HDL subfractions but particularly HDL2-chol concentrations were decreased in RA, primarily in women. This seems to be associated with disease activity and is of clinical relevance. The reduction of the HDL subfraction concentrations, particularly the supposedly beneficial HDL2-chol, may negatively impact the cardiovascular risk profile of women with RA.

## Introduction

Cardiovascular morbidity and mortality are increased in the rheumatoid arthritis (RA) population [1-3]. As much as 50% of all deaths in RA patients can be attributed to cardiovascular events [1]. The risk of cardiovascular disease (CVD) in RA is approximately two- to three-fold greater than in the general population, reaching that of patients with type 2 diabetes mellitus, according to recent studies [4]. As traditional risk factors do not fully account for the increased CVD risk in RA, it can be suggested that inflammation plays an important role in mediating cardiovascular risk in these patients [5,6]. In RA it has been shown that inflammation affects the lipid profile and accelerates atherosclerosis [7,8]. However, it appears that there is no difference in risk of CVD between patients with low or high disease activity [9]. Apparently, low levels of inflammation are sufficient to increase CVD risk in RA.

In the general population, increased levels of total cholesterol (TC), low-density-lipoprotein cholesterol (LDL) and triglycerides, and decreased levels of high-density lipoprotein cholesterol (HDL), that is, a pro-atherogenic lipid profile, are important risk factors for CVD [10]. In the general population, HDL is regarded as the main anti-atherogenic lipoprotein and increased levels of HDL have been associated with a protective effect against cardiovascular mortality and morbidity [11,12]. The beneficial effect of HDL appears to be the strongest for women [12]. This advantageous effect of HDL is supposed to be accomplished primarily through the reverse cholesterol transport (RCT) and the neutralization of oxidized lipids [13].

In RA patients, however, the effect of changes in lipid concentrations on CVD risk in RA patients is less straight forward [8]. Lipoprotein and apolipoprotein levels are known to fluctuate during the course of RA, possibly under the influence of inflammation and anti-inflammatory treatment, including oral steroids and biologic therapies [14-17]. During active disease, increased levels of TC, triglycerides (TG) and apolipoprotein B (ApoB), and reduced concentrations of HDL have been reported [14]. Other aspects of the lipid profile may be of importance. The inflammatory response in RA patients may compromise the beneficial anti-atherogenic effect of HDL on CVD risk. In addition to lower levels of HDL [14,18,19], inflammation may reduce the anti-oxidative capacity, impair RCT capacity of HDL in RA patients, and even lead to HDL becoming pro-inflammatory [20-23]. The functionality of HDL is partially dependent on HDL composition. Based on its density HDL can be divided into two main subfractions: HDL2-cholesterol (HDL2-chol) and the smaller HDL3-cholesterol (HDL3-chol). HDL2-chol has been suggested to be the more variable component of total HDL, while it is higher levels of the HDL2-chol subfraction that contribute to the anti-atherogenic effect of HDL [24-26]. For that reason, decreased levels of HDL subfractions, particularly HDL2-chol, could contribute to the risk of CVD in RA. However, it is currently unclear whether HDL2-chol and HDL3-chol concentrations are actually decreased in RA, and whether their levels are associated with the level of disease activity. Therefore, the objective of this study was to investigate whether HDL2-chol and HDL3-chol concentrations differ between RA patients and healthy controls, and to investigate whether HDL2-chol and HDL3-chol concentrations are associated with the level of RA disease activity.

## Materials and methods

### Study design

This is a cross-sectional study comparing a group of 45 consecutive patients with RA, with a control group of 45 consecutive healthy individuals.

### Patients

RA patients were included in the study if they fulfilled the 1987 American College of Rheumatology classification criteria for RA, did not use lipid-lowering drugs, had no cardiovascular comorbidity (hypertension was allowed) or diabetes. Disease activity scores (DAS28) were registered. The controls were consecutively recruited from healthy blood-donors who came to the regional blood bank for a donation and were matched for gender. The donors did not suffer from any inflammatory or auto-immune disorders and did not use lipid-lowering drugs, and were free of cardiovascular comorbidity and diabetes. All patients and controls who were entered in the study provided their informed consent. The Medical Ethical Committee Arnhem-Nijmegen approved the study.

An *a priori *sample size calculation was deemed inappropriate, as evidence regarding HDL2-chol and HDL3-chol concentrations is limited and no studies have been performed investigating these concentrations in RA patients.

### Lipoprotein measurements

In both patients and controls, non-fasting blood samples (30 ml) were collected, using vacutainer tubes (Beckton Dickinson, Rutherford, New Jersey, USA) containing K3-ethylenediaminetetraacetic acid (EDTA) (1 mg/ml), and a sample was taken in a tube without anticoagulant to obtain serum. Tubes were centrifuged at 3,600 rpm for 10 minutes at 23°C, and frozen at -80°C until assay. Levels of plasma TC, TG and HDL-cholesterol were determined enzymatically on an Eroset Hitachi 747 analyzer which was validated for these particular measurements. During each run, samples from both patients and controls were analyzed to prevent any within and between run variability to affect group differences in HDL subfraction levels. Low-density lipoprotein (LDL) cholesterol levels were calculated according to the Friedewald formula, which provides reliable values up to a triglyceride concentration of 4.0 mmol/L. ApoB and A-I (ApoA) levels (mg/L) were determined by immunonephelometry. HDL subfractions were isolated by means of ultracentrifugation [27]. In short, 2 ml plasma was introduced in MSE (Measuring and Scientific Equipment) tubes of the ultracentrifuge (Beckman L7-55). KBr and CBBR (Coomassie Brilliant Blue R) (1.5% solution) was added and the tubes were centrifuged at 44,000 rpm for 22 hours at 15°C. HDL2-chol (density 1.08 g/ml) and HDL3-chol (density 1.149 g/ml) were separated and their concentrations were measured as previously described for total HDL, correcting for density and volume (2 ml).

### Statistical analyses

The primary outcome was the difference in the concentration of HDL2-chol (mmol/L) between RA patients and the control group. Secondary outcomes were between-group differences in HDL3-chol (mmol/L), HDL2:HDL3 ratio, TC, TG, HDL, LDL-c (mmol/L), ApoA-I and ApoB (mg/L) levels. The differences in HDL2-chol and HDL3-chol concentrations and lipoprotein levels (TC, TG, LDL-c, HDL, ApoA-I and ApoB) between RA patients and healthy controls were analyzed using independent-sample t-tests, with α = 0.05. To analyze the influence of age and gender on HDL2-chol and HDL3-chol concentrations, analysis of covariance was used with HDL2-chol or HDL3-chol concentrations (mmol/L) as the dependent variable, RA (patient or control) as independent variable, and gender and age as covariates and by including an interaction term for gender and presence of RA. To analyze the effect of disease activity on the HDL2-chol and HDL3-chol concentrations, regression analysis was used with HDL2-chol or HDL3-chol concentrations as the dependent variable, disease activity as the independent variable, and regarding age, gender, smoking, BMI, rheumatoid factor positivity and disease duration as potential confounders. Additional analyses were performed to investigate the effect of the inflammatory markers erythrocyte sedimentation rate (ESR; mm/hour) and C-reactive protein (CRP; mg/L) included in the DAS28. Measured values of CRP levels <5 mg/L were set at 0 mg/L. Potential confounders were added stepwise to the model, with a change in the regression coefficient of disease activity of at least 10% as the selection criterion. Mean HDL2-chol and HDL3-chol levels were determined for patients treated with glucocorticosteroids, methotrexate and biologicals separately and compared by means of non-parametric statistics.

## Results

### Patients and controls

A total of 13 men and 32 women with RA were included with a mean ± standard deviation (SD) age of 58 ± 10 years and 60 ± 11 years, respectively. The same number of healthy controls (*n *= 45) were included, 13 men and 32 women with a mean ± SD age of 54 ± 6 years and 55 ± 8 years, respectively. The participants in the control group were on average 5 years younger than the patients in the RA group (Table 1). Patient characteristics are presented in Table 1.

**Table 1:** Characteristics of the study participants at baseline.

	RA patients (*n *= 45)	Healthy controls (*n *= 45)
Age, mean ± SD (years)	60 ± 10.1	55 ± 7.8
Female, n (%)	32 (71)	32 (71)
Rheumatoid factor positive, n (%)	40 (89)	N/A
DAS28, mean ± SD	3.1 ± 1.7	N/A
ESR, median (P25 to P75)	14 (5 to 28.5)	N/A
CRP, median (P25 to P75)	0 (0 to 12)	N/A
Smoking, n (%)	16 (32)	--
BMI, mean ± SD	25 ± (3.3)	--
Anti-rheumatic medication, n (%)	15 (33)	N/A
Methotrexate	22 (49)	N/A
Other DMARD	11 (24)	N.A
Biological DMARD	15 (33)	N/A
Oral glucocorticoids, n (%)	8 (18)	N/A

BMI, body mass index; CRP, C-reactive protein; DAS28, disease activity score (28 joints); DMARD, disease modifying anti-rheumatic drugs; ESR, erythrocyte sedimentation rate; n, number.

### Lipid and lipoprotein patterns in RA and controls

**Figure 1:**
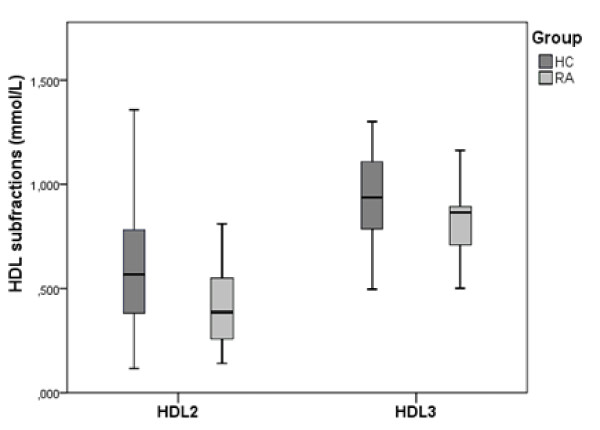
**HDL2-chol and HDL3-chol subfractions in RA patients (RA) and healthy controls (HC)**. The difference in concentrations (mmol/L) in HDL2-chol and HDL3-chol between these groups were compared by means of independent-samples t-test using α = 0.05. Both HDL sub fractions HDL2-chol (*P *= 0.01) and HDL3-chol (*P *= 0.005) were significantly reduced in RA patients. HDL, high density lipoprotein; RA, rheumatoid arthritis.

HDL subfractions HDL2- and HDL3-chol, and other lipid and lipoprotein levels, were compared between the RA and healthy control group. Results are presented in Table 2. The recovery of HDL after separation into subfractions averaged 95%. The results show that TC and LDL levels did not differ significantly, while TG and Apo B levels were significantly higher in RA, and HDL and Apo A-1 levels were lower (Table 2). Notably, both HDL sub fractions HDL2-chol (*P *= 0.01) and HDL3-chol (*P *= 0.005) were significantly reduced in RA patients (Figure 1). Results regarding HDL2-chol concentrations demonstrated a larger difference between RA patients and controls, compared to HDL3-chol, and consequently the HDL2:HDL3 ratio also was significantly lower in RA patients (Table 2).

**Table 2:** Results from the independent-sample t-test of lipid and lipoprotein levels in RA patients and healthy controls.

	RA patients (*n *= 45)	Healthy controls (*n *= 45)	*P*-value
Lipids (mmol/L) TC, mean ± S D	5.7 ± 1.1	5.7 ± 0.8	0.72
TG, mean ± SD	2.1 ± 1.2	1.6 ± 0.8	0.04
HDL, mean ± SD	1.3 ± 0.3	1.6 ± 0.3	<0.001
HDL2-chol	0.5 ± 0.3	0.7 ± 0.4	0.01
HDL3-chol	0.8 ± 0.2	0.9 ± 0.2	0.005
HDL2:HDL3	0.5 ± 0.3	0.7 ± 0.4	0.04
LDL-c, mean ± SD	3.5 ± 0.9	3.3 ± 0.7	0.41
Lipoproteins (mg/L)			
ApoA-I, mean ± SD	1517.2 ± 242.2	1710.1 ± 217.2	<0.001
ApoB, mean ± SD	987.5 ± 220.3	862.2 ± 188.8	0.005

Apo, apolipoprotein; HDL, high density lipoprotein; LDL, low density lipoprotein; n, number; RA, rheumatoid arthritis; SD, standard deviation; TC, total cholesterol; TG, triglycerides.

### Influence of age and gender on HDL2-chol and HDL3-chol concentrations

**Figure 2:**
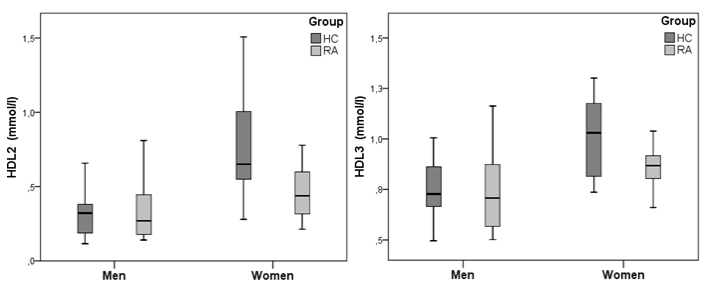
**HDL2-chol and HDL3-chol subfractions in male and female RA patients (RA) and healthy controls (HC)**. The observed differences in mean HDL3-chol and particularly HDL2-chol levels (mmol/L) between RA patients and controls were primarily present in women, not men. In women, the differences between RA patients and healthy controls in HDL2-chol and HDL3-chol were larger; 0.27 mmol/L (95%CI 0.09 to 0.45) for HDL2-chol, and 0.16 mmol/L, (95% CI 0.08 to 0.23) for HDL3-chol. CI, confidence interval; HDL, high density lipoprotein; RA, rheumatoid arthritis.

There appeared to be no association between age and each of the HDL subfractions HDL2-chol and HDL3-chol (not shown). In healthy controls, both HDL2-chol (*P *< 0.001) and HDL3-chol (*P *< 0.001) levels were significantly higher in women. In RA patients these levels were not different between men and women (*P *= 0.13 and *P *= 0.20, respectively). Therefore, the difference between RA and healthy controls was analyzed for men and women separately. In men, the differences between RA and healthy controls in HDL2-chol (0.011 mmol/L, 95% confidence interval (CI) -0.17 to0.15) and HDL3-chol (0.007 mmol/L, 95%CI -0.16 to 0.18) were not significant (*P *= 0.89 and *P *= 0.93, respectively) (Figure 2). For women, these differences between RA patients and controls in HDL2-chol (0.27 mmol/L, 95%CI 0.10 to 0.45) and HDL3-chol (0.16 mmol/L, 95%CI 0.08 to 0.23) were significant (*P *= 0.003 and *P *< 0.001, respectively). After repeating the analyses using linear regression with an interaction term for group and gender (see methods), it appeared that there were interactions regarding gender and presence of RA for HDL2-chol (*P *= 0.055), as well as for HDL3-chol (*P *= 0.063). In women, the HDL2:HDL3 ratio was significantly different between RA and controls, with a mean difference of 0.2 (95% CI 0.04 to 0.40, *P*= 0.02) compared to a mean difference of 0.01 (95% CI -0.2 to 0.1, *P *= 0.9) in men. These findings are mirrored in TG, ApoA-I and Apo B concentrations. TG and Apo B levels were significantly increased only in female RA patients compared to female controls (*P *= 0.002 and *P *= 0.005, respectively). Apo A-1 levels were found to be significantly lower in female RA patients with a mean difference of 229.8 mg/L (*P *< 0.001) and similar in men with a mean difference of 88.6 mg/L (*P *= 0.379).

### HDL2-chol, HDL3-chol and disease activity in RA

**Figure 3:**
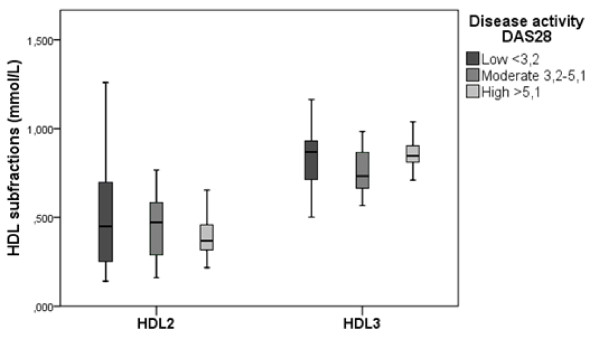
**HDL2-chol and HDL3-chol concentrations in a group of RA patients with low, moderate and high disease activity levels**. HDL2-chol and HDL3-chol concentrations were similar across all categories of the DAS28 (low <3.2; n *= *31, medium 3.2 to 5.1; *n *= 6, and high >5.1; *n *= 8). The results of the regression analysis showed a small effect of DAS28 on HDL2-chol; a decrease in HDL2-chol of 0.06 mmol/L with every point increase in DAS28 (*P *= 0.05). Disease activity did not significantly relate to HDL3-chol, which decreased by 0.02 mmol/L with every point increase in DAS28 (*P *= 0.19). DAS28, disease activity score (28 joints); HDL, high density lipoprotein; n, number; RA, rheumatoid arthritis.

Figure 3 shows that in RA patients the HDL2-chol and HDL3-chol concentrations were similar across all categories of the DAS28 (low <3.2; n *= *31, medium 3.2 to 5.1; *n *= 6, and high >5.1; *n *= 8). The effect of disease activity, as a continuous measure, on HDL2-chol concentrations was estimated using regression analysis, with gender, age, disease duration and use of glucocorticosteroids as covariates, while smoking, BMI and rheumatoid factor were not acting as confounders. A statistically small effect of DAS28 on HDL2-chol was found (Figure 3). HDL2-chol concentration decreased by 0.06 mmol/L with every point increase in DAS28 (*P *= 0.05), while correcting for gender, age, disease duration and use of glucocorticosteroids. Disease activity did not significantly relate to HDL3-chol, which decreased by 0.02 mmol/L with every point increase in DAS28 (*P *= 0.19), while correcting for gender, age, disease duration and use of glucocorticosteroids. The inflammatory marker ESR was significantly associated with HDL2-chol (*P *= 0.046) and HDL3-chol (*P *= 0.006), while correcting for gender, age, disease duration and use of glucocorticosteroids. (CRP did not have a significant effect on either HDL2-chol or HDL3-chol levels (*P *= 0.51 and *P *= 0.21, respectively). The effect on HDL2- and HDL3-chol for every point increase in ESR (mm/hour) and CRP (mg/L) was small; a decrease of 0.006 mmol/L and 0.002 mmol/L, respectively, in HDL2-chol, and a decrease of 0.004 mmol/L and 0.002 mmol/L, respectively, in HDL3-chol.

### HDL2-chol, HDL3-chol in relation to treatment and disease duration in RA

Use of (oral) glucocorticosteroids was included as a confounder in the analysis of the effect of disease activity on HDL2-chol and HDL3-chol concentrations. The mean ± SD HDL2-chol and HDL3-chol levels in users and non-users of methotrexate, biologicals and glucocorticosteroids are presented in Table 3. The subanalysis of HDL2-chol and HDL3-chol levels in non-users and users of glucocorticosteroids (at the time of the study) showed moderately lower mean concentrations of HDL2-chol and moderately higher HDL3-chol levels in users (Table 3), without reaching statistical significance. Glucocorticosteroid use and mean prescribed dosage was similar in men and women (not shown). Disease duration was considered a confounder as well. In patients with a longer disease duration (≥10 years) versus patients with a shorter disease duration (<10 years), higher levels of both HDL2-chol (mean ± SD 0.6 ± 0.4 versus 0.3 ± 0.1) and HDL3-chol (mean ± SD 0.9 ± 0.1 versus 0.7 ± 0.2) concentrations were found. Interestingly, in this study, women had a longer mean ± SD disease duration (18 ± 11 years versus 8 ± 7 years in men) and a higher mean ± SD DAS28 score (3.4 ± 1.8 versus 2.4 ± 1.2).

**Table 3:** Mean HDL2-chol and HDL3-chol levels in RA patients treated with methotrexate, biologicals and glucocorticosteroids at the time of the study.

	Methotrexate			Biologicals			Glucocorticosteroids		
	Yes N = 22	No N = 23	*P*	Yes	No	*P*	Yes	No	*P*
HDL2-chol (mmol/L) mean ± SD	0.47 ± 0.4	0.46 ± 0.3	0.70	0.42 ± 0.2	0.49 ± 0.4	0.83	0.43 ± 0.2	0.48 ± 0.4	0.87
HDL3-chol (mmol/L) mean ± SD	0.82 ± 0.2	0.83 ± 0.2	0.94	0.82 ± 0.2	0.84 ± 0.2	0.36	0.86 ± 0.1	0.81 ± 0.2	0.34

HDL, high density lipoprotein; N, number; RA, rheumatoid arthritis; SD, standard deviation.

## Discussion

This study is the first to investigate the distribution of HDL subfractions in RA patients. According to our results, both HDL subfractions but particularly HDL2-chol concentrations were decreased in RA, leading to a decreased HDL2:HDL3 ratio in these patients. Intriguingly, the differences in HDL subfractions between RA and controls were most evident in women, whereas similar levels of HDL2-chol and HDL3-chol have been observed in men. Disease activity was not strongly related to the level of HDL2-chol or HDL3-chol. Finally, our results suggest that the low HDL2-chol concentration might contribute to the previously reported increased cardiovascular risk in RA women.

Results from previous studies investigating HDL and its anti-atherogenic properties in inflammatory conditions such as RA, indicate that HDL function deteriorates and may even become pro-atherogenic in these patients [20-23]. An inverse relation between HDL3-chol and risk of CVD has been previously reported [28,29]. When comparing HDL subfraction levels in cases with CVD and controls the largest differences were found in HDL2-chol concentrations, and often stronger inverse associations between HDL2-chol and CVD were reported [24-26,30]. This is in accordance with our results; the largest difference between RA patients and controls was found in HDL2-chol concentrations, and, consequently, the HDL2:HDL3 ratio was lower in this group. This fact might translate into an impaired RCT, one of the crucial anti-atherogenic mechanisms involving HDL. The RCT relies on the quantity of both HDL2-chol and HDL3-chol. Several enzymes, including cholesterylester transfer protein (CETP) may affect HDL2-chol and HDL3-chol concentrations, by lowering them [13]. Interestingly, higher CETP concentrations have been previously indicated in RA patients providing a possible explanation for the decreased HDL2-chol levels observed in our study [31]. Lower HDL2-chol levels may impair RCT in these patients, contributing to accelerated atherosclerosis. Nevertheless, future research is necessary to clarify the exact mechanisms responsible for the differences in HDL subfractions between RA patients and controls.

In our study, HDL2-chol and HDL3-chol concentrations were lower in men compared to women in both groups investigated. This is in accordance with previous findings in the general population. Interestingly, the differences in subfraction levels that were found between the RA patients and healthy individuals were only apparent in women, whereas no such differences between male RA patients and healthy controls were seen. It has been previously suggested that the excess risk of CVD in RA is primarily attributable to female RA patients [32,33]. Compared to the general population, the mortality rate in RA is increased in both men and women, but mortality from all cardiac causes is larger in women than in men [34]. Hence, it is tempting to speculate that the excess CVD risk in RA women might be partly due to the relative reduction in the beneficial HDL subfraction HDL2-chol. This is supported by previous findings that show a decrease in HDL and HDL2-chol concentrations in post-menopausal women compared to pre-menopausal women [35], a transition that is reported to induce an increase in the risk of CVD, although modest, supposedly due to a decrease in endogenous estrogen [36-38]. Nevertheless, it is unlikely that serum estrogen decisively contributed to the differences in HDL-2 observed in the present study, as it has been demonstrated to be unaltered in RA women [39]. Alternatively, some differences in RA-related parameters between men and women may have contributed to this result. In line with that, we observed that RA women in this study had longer disease duration and higher mean disease activity. However, there is inconsistent evidence of a certain influence of cumulative disease activity on the lipid profile in RA patients. We have previously shown that disease activity and disease duration are not strongly associated with cardiovascular risk, except perhaps longstanding remission [9,40]. Others, in turn, have indicated that disease duration is associated with accelerated atherogenesis and evidence of increased carotid intima-media wall thickness [41] or presence of carotid plaques [42]. Further research is needed in order to shed more light into this interesting issue.

Our results show that in the RA group, there is a modest association between DAS28 and HDL2-chol concentration and no apparent relationship between DAS28 and HDL3-chol concentrations. For each 1.0 increment of the DAS28, HDL-2chol decreased by 0.06 mmol/L. If disease activity in a patient increases from a very low to a high DAS28 score by 3.1 points, from 2.0 to 5.1, theoretically the HDL2-chol concentration would decrease 0.19 mmol/L. Although on a statistical level these differences did not prove to be significant, it is likely that such an effect, if sustained for a longer period of time, would become clinically relevant [43]. Hence, successful suppression of disease activity by means of treatment may also have a clinically relevant effect on risk of CVD by augmenting HDL2-chol and HDL3-chol levels. Due to the limited number of RA patients who were investigated, we were unable to find consistent differences between the various treatment strategies regarding HDL subfraction levels. This is an interesting issue to pursue in future research.

There are several limitations of this study that require consideration. Data regarding variables that could be possible confounders in the analysis of lipid concentrations, such as smoking and BMI were not available for the healthy controls and were, therefore, not included in the analysis. Although it is unlikely that differences in these variables between RA patients and controls are of sufficient size to yield biased results, patients and controls were matched on age and gender and randomly selected to prevent selection bias. Also, this is a cross-sectional study and, therefore, no long-term effects are reported. Hence, prolonged exposure to high disease activity or remission over time and the possible effect on HDL composition as well as possible changes in HDL composition before and during the course of RA require further research. Further, lipid measurements were performed using non-fasting blood samples, possibly affecting cholesterol levels. However, HDL concentrations are less likely to be affected [44,45].

## Conclusions

In conclusion, levels of HDL and both HDL2-chol and HDL3-chol, but particularly HDL2-chol, were decreased in female RA patients but not in male patients, compared to healthy controls. This also affected the HDL2:HDL3 ratio, which decreased in RA. Disease activity level seems to be related to the level of HDL2-chol and HDL3-chol, albeit in a modest association. This abnormal HDL-subfraction pattern may negatively impact the risk of CVD in RA patients, particularly women. Larger prospective studies would be further necessary in order to test this hypothesis.

## Abbreviations

ApoA-I: apolipoprotein A-I; Apo-B: apolipoprotein B; BMI: body mass index; CETP: cholesterylester transfer protein; CI: confidence interval; CRP: C-reactive protein; CVD: cardiovascular disease; DAS28: disease activity score (28 joints); DMARD: disease-modifying anti-rheumatic drugs; ESR: erythrocyte sedimentation rate; HDL-chol: high-density-lipoprotein cholesterol; HDL2-chol: high-density-lipoprotein 2 cholesterol; HDL3-chol: high-density-lipoprotein 3 cholesterol; LDL-chol: low-density-lipoprotein cholesterol; RA: rheumatoid arthritis; RCT: reversed cholesterol transport; SD: standard deviation; TC: total cholesterol; TG: triglycerides.

## Competing interests

The authors declare that they have no competing interests.

## Authors' contributions

All authors were involved in drafting the article or revising it critically for important intellectual content, and all authors approved the final version to be published. EA had full access to all of the data in the study and takes responsibility for the integrity of the data and the accuracy of the data analysis. JF, CP, EA were involved in study conception and design. EA, CP, and HL acquired the data. Analysis and interpretation of the data was performed by EA, JF, and CP.
